# Dual-Emissive Monoruthenium Complexes of N(CH_3_)-Bridged Ligand: Synthesis, Characterization, and Substituent Effect

**DOI:** 10.3390/ma16206792

**Published:** 2023-10-20

**Authors:** Si-Hai Wu, Zhe Zhang, Ren-Hui Zheng, Rong Yang, Lianhui Wang, Jiang-Yang Shao, Zhong-Liang Gong, Yu-Wu Zhong

**Affiliations:** 1 School of Medicine, Huaqiao University, Quanzhou 362021, China; 2134111028@stu.hqu.edu.cn (Z.Z.); zhengrhsa@163.com (R.-H.Z.); yrr4924@163.com (R.Y.); 2 Beijing National Laboratory for Molecular Sciences, CAS Key Laboratory of Photochemistry, CAS Research/Education Center for Excellence in Molecular Sciences, Institute of Chemistry, Chinese Academy of Sciences, Beijing 100190, China; shaojiangyang@iccas.ac.cn (J.-Y.S.); zhongyuwu@iccas.ac.cn (Y.-W.Z.)

**Keywords:** dual emission, ruthenium, polypyridyl ligand, electrochemistry, photophysics

## Abstract

Three monoruthenium complexes **1**(PF_6_)_2_–**3**(PF_6_)_2_ bearing an N(CH_3_)-bridged ligand have been synthesized and characterized. These complexes have a general formula of [Ru(bpy)_2_(**L**)](PF_6_)_2_, where **L** is a 2,5-di(*N*-methyl-*N*’-(pyrid-2-yl)amino)pyrazine (dapz) derivative with various substituents, and bpy is 2,2′-bipyridine. The photophysical and electrochemical properties of these compounds have been examined. The solid-state structure of complex **3**(PF_6_)_2_ is studied by single-crystal X-ray analysis. These complexes show two well-separated emission bands centered at 451 and 646 nm (Δ*λ*_max_ = 195 nm) for **1**(PF_6_)_2_, 465 and 627 nm (Δ*λ*_max_ = 162 nm) for **2**(PF_6_)_2_, and 455 and 608 nm (Δ*λ*_max_ = 153 nm) for **3**(PF_6_)_2_ in dilute acetonitrile solution, respectively. The emission maxima of the higher-energy emission bands of these complexes are similar, while the lower-energy emission bands are dependent on the electronic nature of substituents. These complexes display two consecutive redox couples owing to the stepwise oxidation of the N(CH_3_)-bridged ligand and ruthenium component. Moreover, these experimental observations are analyzed by computational investigation.

## 1. Introduction

Dual emissions are an interesting fundamental photophysical phenomenon which means that the two excited states of a luminophore with the same or different spin manifold could be emissive simultaneously. Dual emissions of a single-component luminescent material from the excited states with the same spin manifold are not consistent with Kasha’s rule which states that only the lowest energy excited state of a given spin multiplicity is emissive [1,2]. In addition, the dual-emission phenomena of luminescent materials could be affected by unrecognized impurities, isomers, aggregation behavior, or the fluctuation of instruments. However, this kind of intriguing behavior has been found in different kinds of luminescent materials such as organic small molecules, polymers, quantum dots, hybrid lead halides, metal—organic frameworks (MOFs), and transition-metal complexes (TMCs). Related phenomenon can be divided into three categories including dual fluorescence [3,4,5,6,7,8,9,10], dual phosphorescence [11,12,13,14,15,16,17], and dual fluorescence/phosphorescence [18,19,20,21,22,23,24,25]. Compared with luminescent materials with a single emission band, dual-emissive materials have been used in ratiometric sensing (metal ion [26], molecular oxygen [27,28,29,30], protein [31,32], DNA [33,34], and other species [35,36]), white-emitting generation [37,38,39], near-infrared circularly polarized luminescence [40], and multicolor bioimaging [41,42].

Due to the presence of multiple intraligand and charge-transfer excited states, TMCs are good candidates for constructing dual-emissive materials [43,44]. The dual-emissive characteristic of TMCs could be achieved by tuning electronic structures of the ligand [45,46,47,48] or the synthesis of dinuclear bridged complexes with the same or different metal ions [49,50,51]. To date, a number of dual-emissive TMCs have been reported based on monometallic [52,53,54], dimetallic [55,56,57,58], and multimetallic complexes [59,60,61,62]. The dual-emissive multimetallic complexes often demand complicated synthetic procedures with low production yield. Moreover, the existence of an intra/intermolecular interaction or energy transfer process might hinder the observation of dual-emissive properties [63]. In comparison, monometallic complexes possess simpler structures. The structural modification of monometallic complexes may provide a means to design and construct dual-emissive materials with higher production and quantum yields.

**Scheme 1:**
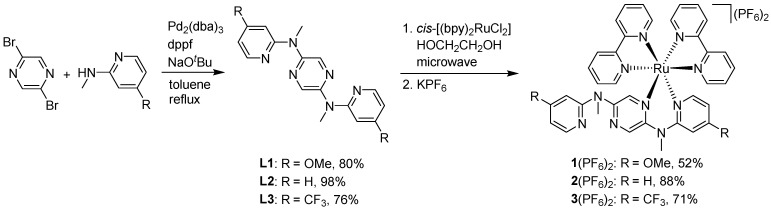
Structures and the synthetic route of complexes **1**(PF_6_)_2_–**3**(PF_6_)_2_.

In our previous work, a fluorescence/phosphorescence dual-emissive mononuclear ruthenium complex [Ru(bpy)_2_(dapz)]^2+^ (**2**^2+^, Scheme 1) was designed and synthesized, where dapz is 2,5-di(*N*-methyl-*N′*-(2-pyridyl)amino)pyrazine, and bpy is 2,2′-bipyridine, respectively [64]. This complex contains an electron-rich bidentate ligand dapz with a large bite angle, and it shows dual emissions as a result of energy-separated excited states. The dual-emissive behavior of **2**(PF_6_)_2_ could be tuned by solvents, oxygen, and metal ions. To extend our work and understand the effect of substituent on dual-emissive behavior, a series of dual-emissive mononuclear ruthenium complexes **1**(PF_6_)_2_–**3**(PF_6_)_2_ have been designed and prepared by changing the electronic nature of the bidentate N(CH_3_)-bridged ligand (Scheme 1).

## 2. Experimental Section

### 2.1. Synthetic Details

All commercial reagents were used directly without further purification. NMR measurements (^1^H and ^13^C NMR) were performed on a Bruker Avance spectrometer in the designated solvents (CD_3_CN and CDCl_3_ for complexes and ligands, respectively). Mass spectrometry data were collected by a Thermo Exactive GC and a Bruker Autoflex III MALDI-TOF mass spectrometer. A Flash EA 1112 analyzer was applied to obtain elemental analysis data.

#### 2.1.1. Synthesis of 2,5-di(N-methyl-N′-(4-methoxy-2-pyridyl)amino)pyrazine (**L1**)

A mixture of 2,5-dibromopyazine (238 mg, 1.0 mmol), *N*-methyl-4-methoxy-2-pyridinamine (304 mg, 2.2 mmol), tris(dibenzylideneacetone)dipalladium(0) (92 mg, 0.1 mmol), sodium tert-butoxide (384 mg, 4.0 mmol), and 1,1′-ferrocenediyl-bis(diphenylphosphine) (55 mg, 0.1 mmol) was dissolved in toluene (10 mL). The reaction mixture was refluxed at 130 °C for two days under an N_2_ atmosphere in a sealed pressure tube. After cooling to room temperature, the solvent was evaporated in vacuo, and the crude product was purified by silica gel column chromatography (eluent: petroleum ether/acetic ether = 5/1) to yield 280 mg of **L1** as a brown solid (80%). ^1^H NMR (400 MHz, CDCl_3_): *δ* = 3.56 (s, 6H), 3.81 (s, 6H), 6.45 (d, *J* = 4.0 Hz, 2H), 6.50 (s, 2H), 8.12 (d, *J* = 4.0 Hz, 2H), 8.44 (s, 2H). ^13^C NMR (100 MHz, CDCl_3_): *δ* = 36.3, 55.4, 97.1, 104.3, 136.4, 148.5, 149.3, 159.2, 167.4. EI-MS (m/z): 352 for [M]^+^. ESI-HRMS: calcd. for C_18_H_20_N_6_O_2_ 352.1648. Found: 352.1645.

#### 2.1.2. Synthesis of 2,5-di(N-methyl-N′-(4-(trifluoromethyl)-2-pyridyl)amino)pyrazine (**L3**)

A suspension of 2,5-dibromopyazine (95 mg, 0.4 mmol), *N*-methyl-4-(trifluoromethyl)-2-pyridinamine (176 mg, 1.0 mmol), tris(dibenzylideneacetone)dipalladium(0) (37 mg, 0.04 mmol), sodium tert-butoxide (153 mg, 1.6 mmol), and 1,1′-ferrocenediyl-bis(diphenylphosphine) (22 mg, 0.04 mmol) was dissolved in toluene (10 mL). The reaction mixture was refluxed at 130 °C for two days under an N_2_ atmosphere in a sealed pressure tube. After cooling to room temperature, the solvent was evaporated in vacuo, and the crude product was purified by silica gel column chromatography (eluent: petroleum ether/acetic ether = 5/1) to yield 130 mg of **L3** as a yellow solid (76%). ^1^H NMR (400 MHz, CDCl_3_): *δ* = 3.65 (s, 6H), 7.03 (d, *J* = 4.0 Hz, 2H), 7.26 (s, 2H), 8.42 (d, *J* = 8.0 Hz, 2H), 8.54 (s, 2H). ^13^C NMR (100 MHz, CDCl_3_): *δ* = 36.4, 107.3, 111.8, 127.3 (q, *J* = 271 Hz), 136.8, 140.7 (q, *J* = 33 Hz), 148.6, 149.4, 157.7. EI-MS (m/z): 428 for [M]^+^. ESI-HRMS: calcd. for C_18_H_14_N_6_F_6_ 428.1184. Found: 428.1190.

#### 2.1.3. Synthesis of **1**(PF_6_)_2_

A suspension of *cis*-[Ru(bpy)_2_Cl_2_] (51.9 mg, 0.1 mmol) and **L1** (35.2 mg, 0.1 mmol) in HOCH_2_CH_2_OH (5 mL) was heated under microwave irradiation for 30 min. The resulting deep red solution was cooled. A saturated KPF_6_ solution was added, and the orange precipitate was formed. A flash column chromatography on SiO_2_ was used to purify the crude product (eluent: CH_3_CN/KNO_3_(aq) = 200/1) to give 55 mg **1**(PF_6_)_2_ as an orange solid in 52% yield. ^1^H NMR (400 MHz, CD_3_CN): *δ* = 3.35 (s, 3H; NCH_3_), 3.44 (s, 3H; NCH_3_), 3.82 (s, 3H; OCH_3_), 3.85 (s, 3H; OCH_3_), 6.38 (s, 1H; OCH_3_–pyridine-H), 6.43 (dd, *J* = 8.0 and 4.0 Hz, 1H; OCH_3_–pyridine-H), 6.56 (dd, *J* = 4.0 and 2.0 Hz, 1H; OCH_3_–pyridine-H), 6.71 (d, *J* = 4.0 Hz, 1H; OCH_3_–pyridine-H), 7.15 (t, *J* = 8.0 Hz, 2H; bipyridine-H), 7.29 (t, *J* = 4.0 Hz, 1H; OCH_3_–pyridine-H), 7.56–7.64 (m, 6H; bipyridine-H), 7.87 (t, *J* = 8.0 Hz, 1H; bipyridine-H), 7.92 (t, *J* = 8.0 Hz, 1H; bipyridine-H), 8.10 (t, *J* = 8.0 Hz, 1H; bipyridine-H), 8.15 (t, *J* = 8.0 Hz, 1H; bipyridine-H), 8.29 (s, 1H; bipyridine-H), 8.33–8.36 (m, 3H; bipyridine-H), 8.40 (d, *J* = 8.0 Hz, 1H; OCH_3_–pyridine-H), 8.49 (d, *J* = 8.0 Hz, 1H; pyrazine-H), 8.58 (d, *J* = 4.0 Hz, 1H; pyrazine-H). ^13^C NMR (100 MHz, CD_3_CN): *δ* = 41.0, 57.2, 101.4, 106.6, 109.3, 124.9, 125.0, 125.1, 125.4, 127.4, 128.0, 128.3, 135.9, 137.1, 138.3, 138.5, 138.8, 138.9, 152.1, 152.6, 152.8, 153.5, 154.1, 158.0, 158.1, 158.2, 169.2. MALDI-MS: *m*/*z* = 910.8 for [M − PF_6_]^+^, 765.9 for [M − 2PF_6_]^2+^, 609.9 for [M − 2PF_6_ − bpy]^2+^. Anal. calcd. for C_38_H_36_F_12_N_10_O_2_P_2_Ru·H_2_O: C, 42.50; H, 3.57; N, 13.04. Found: C, 42.17; H, 3.11; N, 12.96.

#### 2.1.4. Synthesis of **3**(PF_6_)_2_

A suspension of *cis*-[Ru(bpy)_2_Cl_2_] (51.9 mg, 0.1 mmol) and **L3** (42.8 mg, 0.1 mmol) in HOCH_2_CH_2_OH (5 mL) was heated under microwave irradiation for 30 min. The resulting deep red solution was cooled. A saturated KPF_6_ solution was added, and the orange precipitate was formed. A flash column chromatography on SiO_2_ was used to purify the crude product (eluent: CH_3_CN/KNO_3_(aq) = 300/1) to give 80 mg **3**(PF_6_)_2_ as an orange solid in 71% yield. ^1^H NMR (400 MHz, CD_3_CN): *δ* = 3.40 (s, 3H; NCH_3_), 3.57 (s, 3H; NCH_3_), 7.03 (d, *J* = 8.0 Hz, 1H; CF_3_–pyridine-H), 7.13 (d, *J* = 4.0 Hz, 1H; CF_3_–pyridine-H), 7.20–7.24 (m, 2H; CF_3_–pyridine-H), 7.33 (t, *J* = 8.0 Hz, 1H; bipyridine-H), 7.55–7.67 (m, 6H; bipyridine-H), 7.80 (s, 1H; bipyridine-H), 7.89 (d, *J* = 4.0 Hz, 1H; CF_3_–pyridine-H), 7.93 (t, *J* = 8.0 Hz, 1H; bipyridine-H), 7.98 (t, *J* = 8.0 Hz, 1H; bipyridine-H), 8.17 (t, *J* = 8.0 Hz, 1H; bipyridine-H), 8.21 (t, *J* = 8.0 Hz, 1H; bipyridine-H), 8.36–8.41 (m, 4H; bipyridine-H), 8.48 (d, *J* = 8.0 Hz, 1H; CF_3_–pyridine-H), 8.52–8.55 (m, 2H; pyrazine-H). ^13^C NMR (100 MHz, CD_3_CN): *δ* = 36.1, 41.4, 108.9, 112.9, 113.7, 116.4, 122.0, 122.6, 124.7, 125.2, 125.5, 125.6, 127.9, 128.2, 128.3, 128.5, 136.2, 138.8, 138.9, 139.3, 139.4, 140.3 (q, *J* = 61.6 Hz), 148.1, 149.7, 149.9, 152.6, 152.7, 153.6, 153.7, 153.9, 157.6, 158.0, 158.2, 160.6. MALDI-MS: *m*/*z* = 986.7 for [M − PF_6_]^+^, 842.8 for [M − 2PF_6_]^2+^, 684.8 for [M − 2PF_6_ − bpy]^2+^. Anal. calcd. for C_38_H_30_F_18_N_10_P_2_Ru: C, 40.33; H, 2.67; N, 12.38. Found: C, 40.23; H, 2.84; N, 12.17.

### 2.2. X-ray Crystallography

The X-ray diffraction measurements were performed on a Rigaku Saturn 724 diffractometer on a rotating anode (Mo Kα radiation, *λ* = 0.71073 Å). Olex2 software (SVN Revision No. 5506) was applied to analyze the data and generate the structure graphic. The crystallographic data of **3**(PF_6_)_2_ (CCDC 2107266) were deposited in The Cambridge Crystallographic Data Centre for free.

### 2.3. Spectroscopic Measurements

The electronic absorption measurements were performed on a TU-1810DSPC spectrometer in chromatographic grade acetonitrile at room temperature. An F-380 spectrofluorometer was employed to collect the steady-state emission and excitation spectra. The samples of photophysical analysis were prepared by using the 1 cm pathlength quartz cuvettes. The quinine sulfate in 1.0 M aq H_2_SO_4_ (*Φ* = 55%) and [Ru(bpy)_3_](PF_6_)_2_ (*Φ* = 9.5%) were used to calculate the relative luminescence quantum yields in degassed CH_3_CN as the reference. The luminescence decays and temperature-dependent emission studies were carried out on the FLS920 and FLS1000 spectrophotometer, respectively.

### 2.4. Electrochemical Measurements

All electrochemical measurements were carried out on a CHI660D electrochemical station based on a three-electrode system. After bubbling with nitrogen for 5 min, the samples of electrochemical studies were tested at a scan rate of 100 mV/s in 0.1 M Bu_4_NClO_4_/CH_3_CN. The potential of a saturated Ag/AgCl electrode was used to calculate the relative potentials of these compounds as the reference.

### 2.5. DFT and TDDFT Calculations

The B3LYP exchange-correlation functional and the Gaussian 09 software package were employed for the density functional theory (DFT) calculations. The crystallographic data of **3**(PF_6_)_2_ were employed to generate the input files. The Los Alamos effective core potential LANL2DZ basis set for Ru and 6-31G* for other atoms were used to optimize the electronic structures of these compounds. All of the calculations were carried out including the solvation effects and using the no symmetry constraints. The DFT-optimized structures were employed for the TDDFT calculations on the same level of theory. In order to make the optimized geometries be local minima, frequency calculations were carried out on the same level of theory. An isovalue of 0.02 e bohr^−3^ was set for all orbitals on calculations.

### 2.6. HPLC Analysis

All HPLC measurements were performed on a Shimadzu UFLC system. The analysis data were collected by a Shim-pack XR-ODS column (2.2 μm, 75 mm × 4.6 mm, i.d.). A gradient solvent of CH_3_CN in water was used to elute these samples (10−90% over 0–10 min, followed by an isocratic elution of 90% CH_3_CN for 5 min). A 0.1% of trifluoroacetic acid was added in all solvents. The flow rate was set at 1.0 mL/min. The detection wavelengths were set at 450, 451, and 430 nm for **1**(PF_6_)_2_, **2**(PF_6_)_2_, and **3**(PF_6_)_2_, respectively.

## 3. Results and Discussions

### 3.1. Studies on Preparation and Single-Crystal X-ray Analysis

Three N(CH_3_)-bridged ligands **L1**–**L3** and corresponding mononuclear ruthenium complexes **1**(PF_6_)_2_–**3**(PF_6_)_2_ were synthesized as outlined in Scheme 1. The bidentate ligands **L1**–**L3** were obtained through a Pd-catalyzed C-N coupling reaction of 2,5-dibromopyrazine with 2-(*N*-methylamino)pyridine derivatives in the range of 76−98% yields [64,65]. The reaction of **L1**, **L2**, and **L3** with 1 equiv. *cis*-[Ru(bpy)_2_Cl_2_] under microwave irradiation, followed by anion exchange using potassium hexafluorophosphate, provided complexes **1**(PF_6_)_2_, **2**(PF_6_)_2_, and **3**(PF_6_)_2_ in a 52%, 88%, and 71% yield, respectively. These new compounds were fully characterized by nuclear magnetic resonance (NMR) (Figures S1–S8), mass spectrometry (Figures S9–S11), and elemental analysis. Furthermore, high performance liquid chromatography (HPLC) analysis results indicate that these complexes have high purities (Figure S12).

**Figure 1:**
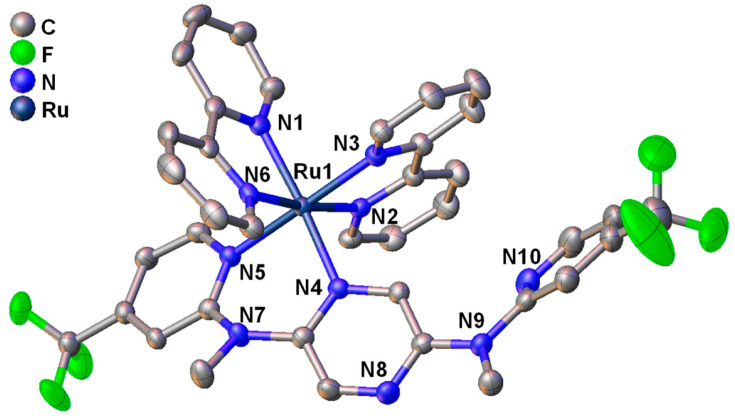
The crystal structure of complex **3**(PF_6_)_2_. The thermal ellipsoids are set at 50% probability. PF^−^_6_, solvents, and H atoms excluded for clarity.

The solid-state structure of complex **3**(PF_6_)_2_ was determined by single-crystal X-ray analysis. Figure 1 shows the ORTEP diagram, and Tables S1 and S2 in the Supporting Information summarize the crystallographic data. A single crystal of complex **3**(PF_6_)_2_ was acquired by the slow diffusion of (C_2_H_5_)O into a solution of the complex in CH_3_CN. The coordination geometry of the ruthenium atom has a distorted octahedral with bpy and **L3**. The N–Ru–N bite angle of the bpy (78.86(11)° and 78.68(11)°) is smaller than that of ligand **L3** (88.30(10)°). Similar findings have been recorded in our previously reported N^∧^N bidentate Ru(II) complexes [64,65]. The Ru–N bond lengths of complex **3**(PF_6_)_2_ are in the range of 2.056(3)−2.099(3) Å. No distinct length difference is present among the Ru–N bonds associated with bpy and **L3**. The bidentate ligand bpy has a planar structure. However, ligand **L3** shows a severely twisted structure. The torsion angles between the two pyridine planes and the pyrazine plane of **L3** are 26.38° and 127.73°, respectively.

### 3.2. Spectroscopic Studies

**Figure 2:**
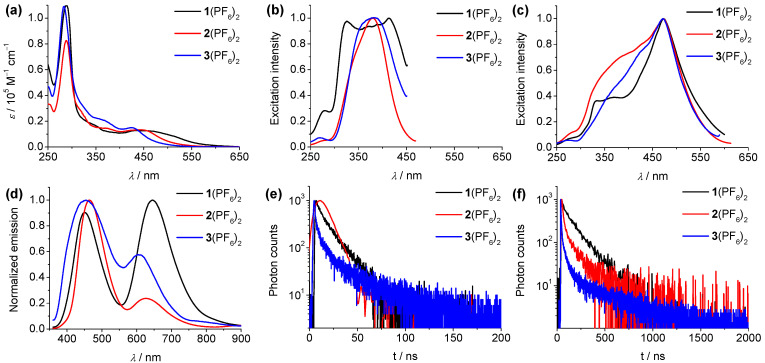
(**a**) UV/vis absorption spectra of complexes **1**(PF_6_)_2_–**3**(PF_6_)_2_ in acetonitrile at a concentration of 5 × 10^−5^ M. (**b**,**c**) Excitation spectra of complexes **1**(PF_6_)_2_–**3**(PF_6_)_2_ at higher- and lower-energy emission bands. (**d**) Emission spectra of complexes **1**(PF_6_)_2_–**3**(PF_6_)_2_ in acetonitrile on excitation at 350 nm. (**e**,**f**) Emission decay profiles of complexes **1**(PF_6_)_2_–**3**(PF_6_)_2_ at higher- and lower-energy emission bands under N_2_-saturated condition.

The absorption, excitation, and steady-state emission spectra and the emission decay studies of complexes **1**(PF_6_)_2_–**3**(PF_6_)_2_ in acetonitrile are shown in Figure 2, and their photophysical data are summarized in Table 1. Ligands **L1**–**L3** display two absorption bands at 310 and 360 nm, 310 and 361 nm, and 295 and 353 nm, respectively (Figure S13). These absorption bands in the UV and visible region are ascribed to the *π*–π* excitations and the intraligand charge-transfer (ICT) transitions from the amine unit to pyrazine ring of the N(CH_3_)-bridged ligand, respectively. Under nitrogen saturated conditions, ligands **L1**–**L3** display structureless emission bands at 457, 445, and 450 nm with quantum yields of 24%, 43%, and 0.06%, respectively, relative to 55% of quinine sulfate in 1.0 M aq H_2_SO_4_ (Figure S13). In the nitrogen saturated solutions, the excited-state lifetimes of these emission bands were determined to be 5.6, 11.5, and 0.4 ns for **L1**–**L3** (Table 1), respectively. These results indicate that the substituent effect will increase the non-emissive deactivation pathway of these ligands and thus impair the emission properties. Compared with **L3** with electron-withdrawing –CF_3_ groups, **L1** with electron-donating –OMe groups shows better quantum yield and longer excited-state lifetime due to the inductive effect of an alkoxyl substituent [66,67]. These experimental findings indicate that these emission bands of ligands **L1**–**L3** are of singlet charge-transfer (^1^CT) character.

Complexes **1**(PF_6_)_2_–**3**(PF_6_)_2_ show intense absorption bands in the UV region at 289, 288, and 284 nm, respectively (Figure 2a), which are assigned to *π*–π* transitions of ligands. The intense and broad bands in the visible region are observed at 350 and 467 nm, 375 and 448 nm, and 368 and 427 nm for **1**(PF_6_)_2_–**3**(PF_6_)_2_, respectively. As indicated by time-dependent density functional theory (TDDFT) calculations below, these higher-energy absorption bands are associated with the ligand-to-ligand charge-transfer (LLCT) transitions from the N(CH_3_)-bridged ligand to bpy and ICT of the N(CH_3_)-bridged ligand, while the lower-energy absorption bands are assigned to the metal-to-ligand charge-transfer (MLCT) transitions from the ruthenium component to bpy ligand. By variation of the substituents, **3**(PF_6_)_2_, with electron-withdrawing –CF_3_ groups on the pyridine rings shows a 40 nm blue shift of the MLCT absorption band in comparison to **1**(PF_6_)_2_ containing electron-donating –OMe groups. All three complexes show intriguing dual-emissive behavior in dilute acetonitrile solution under irradiation, with two well-separated emission bands centered at 451 and 646 nm for **1**(PF_6_)_2_, 465 and 627 nm for **2**(PF_6_)_2_, and 455 and 608 nm for **3**(PF_6_)_2_, respectively. The wavelength difference between the dual emission maxima (Δ*λ*_max_) is 195, 162, and 153 nm for **1**(PF_6_)_2_–**3**(PF_6_)_2_, respectively (Figure 2d). The excitation spectra of the higher-energy emission bands of these complexes are associated with the LLCT/ICT absorptions, while the longer-wavelength MLCT absorptions are basically in accordance with the excitation spectra of the lower-energy emission bands with a slight red shift (Figure 2b,c and Figure S14). The emission maxima of the higher-energy emission bands of these complexes have similar emission wavelength, while the lower-energy emission band shows a 38 nm blue shift from **1**(PF_6_)_2_ to **3**(PF_6_)_2_ with decreasing electron-donating capabilities of substituents. This is consistent with an ascending order of the energy of the MLCT absorption band from **1**(PF_6_)_2_ to **3**(PF_6_)_2_. The solvent effect on the dual-emissive properties of **2**(PF_6_)_2_ was reported in our previous work [64]. Similar results were observed for **1**(PF_6_)_2_ and **3**(PF_6_)_2_. The relative intensities and emission maxima of these two emission bands are also strongly dependent on the solvent polarity. For instance, the lower-energy emission band of **1**(PF_6_)_2_ became dominant in DMF, while the two emission bands exhibited comparable intensities in other solvents. For complex **3**(PF_6_)_2_, the higher-energy emission band became dominant in CH_3_CN, acetone, and CH_3_OH, while the lower-energy emission band displayed a much higher intensity in DMSO. In DMF, the two emission bands have comparable intensities (Figure S15). The emission spectra in the solid state of **1**(PF_6_)_2_ and **3**(PF_6_)_2_ exhibit similar emission maxima (600 and 645 nm for **1**(PF_6_)_2_ and **3**(PF_6_)_2_, respectively) with respect to that of the lower-energy emission band in CH_3_CN (Figure S16). Compared with the lower-energy emission band in CH_3_CN, the emission spectrum of pure powder of **2**(PF_6_)_2_ shows a 31 nm red shift most likely due to the aggregates formed by intermolecular interaction. In nitrogen saturated acetonitrile solution, complex **3**(PF_6_)_2_ has a low quantum yield of 0.53%, relative to 9.5% of [Ru(bpy)_3_](PF_6_)_2_ [68], while complex **1**(PF_6_)_2_ shows a better quantum yield of 2.1%. Compared with the prototype complex **2**(PF_6_)_2_, complexes **1**(PF_6_)_2_ and **3**(PF_6_)_2_ possess lower emission quantum yields. We conjecture that the introduction of substituents may increase the flexibility of structure and molecular vibrations and enhance the rate constant of the nonradiative process.

In luminescence decay studies, all three complexes **1**(PF_6_)_2_–**3**(PF_6_)_2_ exhibit distinct excited-state lifetimes for two emission bands in acetonitrile solution (Figure 2e,f, and Table 1). In air-equilibrated solutions, the excited-state lifetimes of the higher-energy emission bands are 14, 10, and 10 ns, while the lower-energy emission bands have longer lifetimes of 89, 58, and 24 ns for **1**(PF_6_)_2_–**3**(PF_6_)_2_, respectively. In nitrogen saturated solutions, the lifetimes of the higher-energy emission bands show little changes with respect to those in air-equilibrated solutions (16, 13, and 15 ns for **1**(PF_6_)_2_–**3**(PF_6_)_2_, respectively), while the lifetimes of the lower-energy emission bands are considerably elongated (193, 219, and 189 ns for **1**(PF_6_)_2_–**3**(PF_6_)_2_, respectively). Compared with ligands, the substituent effect on excited-state lifetimes of these complexes is negligible. Moreover, the lifetimes of the representative complexes **1**(PF_6_)_2_ and **2**(PF_6_)_2_ were measured in glassy CH_3_CN at 77 K. The higher-energy emission bands still exhibit nanosecond range lifetimes (5.0 ns for both **1**(PF_6_)_2_ and **2**(PF_6_)_2_), and those of the lower-energy emission bands significantly increase to a microsecond range (0.53 and 2.5 μs for **1**(PF_6_)_2_ and **2**(PF_6_)_2_, respectively). Based on these experimental observations, the higher- and the lower-energy emission bands are ascribed to the admixtures of the singlet (N(CH_3_)-bridged ligand to bpy ^1^LLCT and N(CH_3_)-bridged ligand ^1^ICT charge transfer) and the triplet ^3^MLCT (ruthenium component to bpy charge transfer) character, respectively. A number of studies suggest that the dual fluorescence/phosphorescence character of a single complex was found in different luminescent TMCs such as platinum [18,19,20,27,29], osmium [21], copper [22], and iridium [24,30], as well as ruthenium complexes [28,32,34]. Kasha’s rule states that only the lowest energy excited state of a given spin multiplicity is emissive. However, the dual emissions coming from the excited states with the different spin manifold of a single complex are still obeying Kasha’s rule.

### 3.3. Temperature-Dependent Emission Spectral Studies

**Figure 3:**
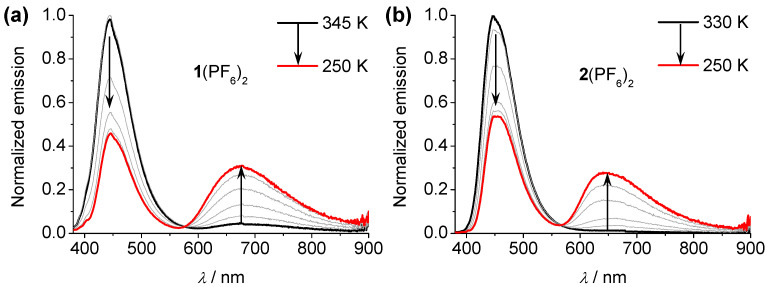
Temperature-dependent emission spectra changes of complexes **1**(PF_6_)_2_ (**a**) and **2**(PF_6_)_2_ (**b**) in CH_3_CN on excitation at 350 nm.

To investigate the changes in dual-emissive properties by temperature stimuli, the temperature-dependent emission spectral studies of the representative complexes **1**(PF_6_)_2_ and **2**(PF_6_)_2_ have been measured in CH_3_CN solution and shown in Figure 3. Both of the complexes display similar emission spectral changes. Upon decreasing the temperature from 345 to 250 K, the higher-energy emission intensities decrease significantly, while those of the lower-energy emissions gradually increase. During the spectral changes process, a well isoluminescence point was recorded at 576 and 566 nm for **1**(PF_6_)_2_ and **2**(PF_6_)_2_, respectively. The excited-state lifetimes of the higher-energy emission bands of these complexes still fall in the nanosecond range from 345 to 250 K, excluding a thermally activated process. This suggests that the Franck–Condon transitions of the higher-energy emission are slowed down upon decreasing the temperature and thus facilitate the intersystem crossing from ^1^LLCT/^1^ICT to the ^3^MLCT state [69]. The relative intensity of the two emission bands of **1**(PF_6_)_2_–**3**(PF_6_)_2_ vary as a function of the excitation wavelength (Figure S17). When excited at a shorter wavelength (300–360 nm), the higher-energy emission band is much higher than the lower-energy emission band. When a longer excitation wavelength was applied (380–500 nm), the higher-energy emission band almost disappeared, and the lower-energy emission band became dominant.

### 3.4. Electrochemical Studies

**Figure 4:**
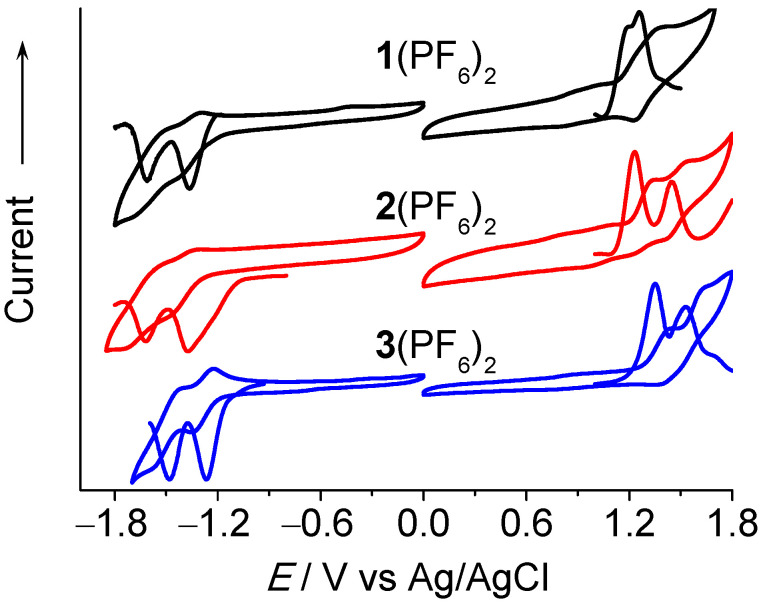
Cyclic and differential pulse voltammograms of **1**(PF_6_)_2_–**3**(PF_6_)_2_ at a scan rate of 100 mV/s in 0.1 M Bu_4_NClO_4_/CH_3_CN.

Cyclic voltammetry (CV) and differential pulse voltammetry (DPV) were applied to study the electrochemical properties of complexes **1**(PF_6_)_2_–**3**(PF_6_)_2_ and ligands **L1**–**L3**. Figure 4 displays the CV and DPV profiles of **1**(PF_6_)_2_–**3**(PF_6_)_2_**,** and their electrochemical data are collected in Table 1, together with ligands **L1**–**L3**. In the anodic scan, **1**(PF_6_)_2_–**3**(PF_6_)_2_ display an irreversible and a reversible oxidation peak at +1.12 and +1.26 V, +1.23 and +1.44 V, and +1.35 and +1.53 V, versus Ag/AgCl in CH_3_CN, respectively. The presence of the electron-donating groups –OMe in complex **1**(PF_6_)_2_ make both oxidation processes more negative in comparison to **2**(PF_6_)_2_ (110 and 180 mV shift for the first and second oxidation peaks, respectively). In contrast, 120 and 90 mV positive redox shifts are observed for the first and second oxidation peaks of **3**(PF_6_)_2_ relative to that of **2**(PF_6_)_2_ due to the introduction of electron-withdrawing –CF_3_ groups. The first irreversible peaks are assigned to the N(CH_3_)-bridged ligand oxidation while the second reversible signals are ascribed to the Ru^II/III^ process. In the cathodic scan, **1**(PF_6_)_2_–**3**(PF_6_)_2_ show two consecutive reversible reduction waves at −1.36 and −1.61 V, −1.37 and −1.62 V, and −1.27 and −1.48 V, versus Ag/AgCl in CH_3_CN, respectively. These peaks are associated with the reductions of bpy ligands. These assignments are consistent with the electrochemical findings of these ligands (Figure S18). **L1**–**L3** display the amine-based irreversible oxidation peaks at +0.84, +0.89, and +1.01 V, versus Ag/AgCl in CH_3_CN, respectively. No obvious reduction processes were found for these ligands. These assignments are also supported by the DFT calculation results discussed below. The difference between the first anodic and first cathodic electrochemical potential (Δ*E*_echem_) of these complexes was employed to calculate the energy gaps which were determined to be 2.48, 2.60, and 2.62 eV for **1**(PF_6_)_2_–**3**(PF_6_)_2_, respectively.

### 3.5. DFT and TDDFT Calculations

**Figure 5:**
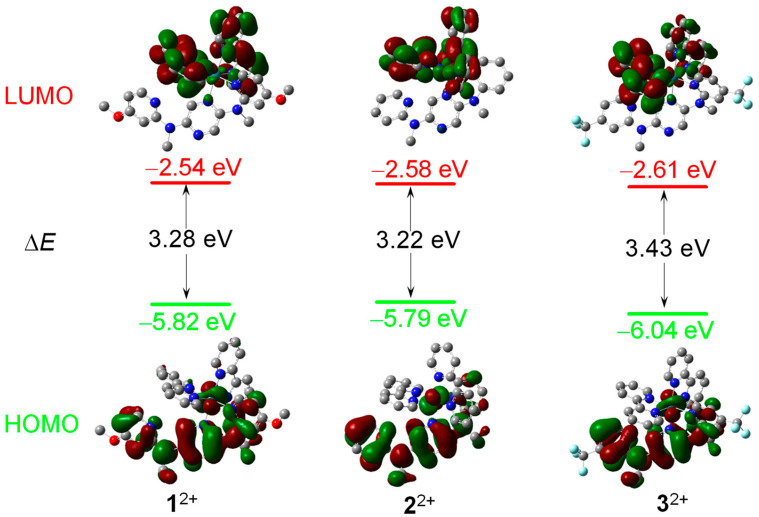
Isodensity plots and energies of HOMO and LUMO of **1**^2+^, **2**^2+^, and **3**^2+^ (isovalue = 0.02 e bohr^−3^).

In order to further understand the electronic transitions of complexes **1**(PF_6_)_2_–**3**(PF_6_)_2_, DFT calculations have been carried out on complexes **1**^2+^, **2**^2+^, and **3**^2+^. The optimized geometries were generated from the single-crystal structure of **3**(PF_6_)_2_. Figure 5 displays the calculated energy diagram and isodensity plots of **1**^2+^−**3**^2+^. The frontier energy gap of complex **3**^2+^ (3.43 eV) is slightly larger with respect to those of **1**^2+^ and **2**^2+^ (3.28 and 3.22 eV, respectively). This is mainly caused by the stabilization of the highest occupied molecular orbital (HOMO) level of **3**^2+^. These calculation findings are in agreement with the above experimental results.

Figures S19 and S20 display the representative frontier orbital isodensity plots of **1**^2+^ and **3**^2+^, respectively. The HOMOs of these complexes are dominated by the N(CH_3_)-bridged ligand, with minor contributions from the ruthenium component. The lower occupied orbitals (HOMO–1, HOMO–2, and HOMO–3) are dominated by the ruthenium ion. The lowest unoccupied molecular orbital (LUMO) and LUMO+1 of these complexes have a bpy character, while the LUMO+2 is dominated by the N(CH_3_)-bridged ligand.

In the same level of theory, TDDFT calculations were performed on the basis of the DFT-optimized structures of these complexes. These predicted electronic transitions are summarized in Table 2 and shown in Figure S21. The predicted *S*_1_ excitation of **1**^2+^ is associated with the HOMO → LUMO transition, which is associated with the low-energy absorption extending over 650 nm. The lower-energy emission band at 646 nm was assigned to the predicted *S*_2_ excitation (HOMO–1 → LUMO+1) which is attributed to the ML_bpy_CT transitions. The higher-energy *S*_3_, *S*_4_, *S*_7_, and *S*_8_ transitions have similar ML_bpy_CT characters. The predicted *S*_9_, *S*_10_, *S*_11_, *S*_12_, *S*_13_, and *S*_19_ excitations are mainly responsible for the observed absorption bands from 330 to 400 nm. TDDFT calculation results indicate that these excitations are associated with LLCT (amine unit of **L1** to bpy), ICT (amine unit to pyrazine ring of **L1**), and **L1**-targeted MLCT transitions. These states are perturbed by MLCT states [64], and they are associated with the higher-energy emission band, indicating the admixtures of the LLCT/ICT character of the excited state. A similar situation was observed for **3**^2+^. The predicted *S*_1_ excitation of **3**^2+^ has a very low oscillator strength (*f*). The lower-energy emission band at 608 nm was assigned to the predicted *S*_2_ excitation (HOMO–1 → LUMO+1) which is attributed to the ML_bpy_CT transitions. The predicted *S*_7_, *S*_9_, *S*_10_, *S*_13_, and *S*_16_ excitations are mainly responsible for the observed higher-energy charge-transfer absorption bands from 350 to 400 nm. These excitations are attributed to LLCT (amine unit of **L3** to bpy), ICT (amine unit to pyrazine ring of **L3**), and **L3**-targeted MLCT transitions, and they are responsible for the observed higher-energy emission band at 455 nm.

## 4. Conclusions

In summary, three monoruthenium complexes **1**(PF_6_)_2_–**3**(PF_6_)_2_ with dual fluorescence/phosphorescence are prepared and characterized. These complexes show well-separated dual emissions that are ascribed to the ^1^LLCT/^1^ICT and ^3^MLCT transitions, respectively. The substituent and solvent effects in the dual-emissive properties of these complexes have been examined. The energy gaps of two emissions can be tuned by introducing different substituents to the N(CH_3_)-bridged ligand, which are decreased by enhancing the electron-withdrawing capabilities of substituents. The relative intensities and emission maxima of two emissions are also strongly dependent on the solvent used. Future work will focus on the design and application of dual-emissive transition-metal complexes as ratiometric photoluminescent probes for the detection of proteins and nucleic acids.

## Figures and Tables

**Table 1:** Photophysical and electrochemical data *^a^*.

Compound	*λ*_max,abs_ [nm] (*ε* [10^5^ M^−1^cm^−1^]) *^b^*	*λ*_max,emi_ [nm] *^c^*	*τ* [ns] *^d^* (air)	*τ* [ns] (N_2_)	*τ* [ns] (77K)	*Φ* (N_2_) *^e^*	*E*_1/2,anodic_ [V] *^f^*	*E*_1/2,cathodic_ [V]
**L1**	310 (0.16), 360 (0.07)	457	ND	5.6	ND	24%	+0.84	ND
**L2**	310 (0.13), 361 (0.07)	445	ND	11.5	ND	43%	+0.89	ND
**L3**	295 (0.08), 329 (0.07), 353 (0.07)	450	ND	0.4	ND	0.06%	+1.01	ND
**1**(PF_6_)_2_	289 (1.10), 350 (0.16), 467 (0.12)	451/646	14/89	16/193	5.0/528	2.1%	+1.12, +1.26	−1.36, −1.61
**2**(PF_6_)_2_	288 (0.83), 375 (0.14), 448 (0.13)	465/627	10/58	13/219	5.0/2500	6.7%	+1.23, +1.44	−1.37, −1.62
**3**(PF_6_)_2_	284 (1.09), 368 (0.21), 427 (0.15)	455/608	10/24	15/189	ND	0.53%	+1.35, +1.53	−1.27, −1.48

*^a^* All spectral data were collected in a 1.0 cm quartz cell. ND = not determined. *^b^* The absorption spectra were measured in acetonitrile. *^c^* The excitation wavelength is 360 nm and 350 nm for ligands and complexes, respectively. *^d^* The data measured were well simulated by a biexponential decay, the average lifetime *τ* was determined by *τ* = [*A*_1_(*τ*_1_)^2^ + *A*_2_(*τ*_2_)^2^]/(*A*_1_*τ*_1_ + *A*_2_*τ*_2_). *^e^* Quantum yield was determined by using the quinine sulfate in 1.0 M aq H_2_SO_4_ (55%) and [Ru(bpy)_3_](PF_6_)_2_ (9.5%) as the reference for ligands and complexes, respectively. *^f^* All data were recorded at a scan rate of 100 mV/s in 0.1 M Bu_4_NClO_4_/CH_3_CN. The *E*_1/2_ value was determined by Ag/AgCl as the reference.

**Table 2:** TDDFT results *^a^*.

Comp.	*S_n_*	*E* [ev]	*λ* [nm]	*f*	Dominant Transition(s) (% Contribution *^b^*)	Assignment *^c^*
**1** ^2+^	1	2.65	468	0.0111	HOMO → LUMO (61)	L_dapz-OMe_L_bpy_CT
	2	2.72	455	0.0190	HOMO–1 → LUMO+1 (41)	ML_bpy_CT
	3	2.73	454	0.0144	HOMO–2 → LUMO (36)	ML_bpy_CT
	4	2.81	442	0.0378	HOMO–2 → LUMO+1 (36)	ML_bpy_CT
	7	3.04	409	0.0678	HOMO–3 → LUMO (34)	ML_bpy_CT
	8	3.11	399	0.0702	HOMO–3 → LUMO+1 (28), HOMO–3 → LUMO (17)	ML_bpy_CT
	9	3.19	389	0.0319	HOMO → LUMO+2 (75)	ICT
	10	3.33	373	0.0150	HOMO–2 → LUMO+2 (59), HOMO–1 → LUMO+2 (22)	ML_dapz-OMe_CT/ICT
	11	3.45	359	0.0596	HOMO–1 → LUMO+2 (39)	ML_dapz-OMe_CT/ICT
	12	3.53	351	0.0696	HOMO–3 → LUMO+2 (69), HOMO–1 → LUMO+2 (16)	ML_dapz-OMe_CT/ICT
	13	3.56	349	0.0102	HOMO → LUMO+3 (18)	L_dapz-OMe_L_bpy_CT
	19	3.81	325	0.0183	HOMO → LUMO+5 (52)	L_dapz-OMe_L_bpy_CT
**3** ^2+^	2	2.81	441	0.0107	HOMO–1 → LUMO+1 (35)	ML_bpy_CT
	3	2.83	438	0.0181	HOMO–1 → LUMO (23), HOMO–1 → LUMO+1 (27)	ML_bpy_CT
	4	2.93	423	0.0467	HOMO → LUMO+1 (40)	L_dapz-CF3_L_bpy_CT
	7	3.13	396	0.0930	HOMO–3 → LUMO (50)	ML_bpy_CT
	9	3.17	391	0.0555	HOMO–2 → LUMO+1 (37)	ML_bpy_CT
	10	3.27	379	0.0406	HOMO–1 → LUMO+3 (26), HOMO → LUMO+3 (33)	ML_dapz-CF3_CT/ICT
	13	3.42	363	0.0945	HOMO–2 → LUMO+2 (67), HOMO → LUMO+2 (14)	ML_dapz-CF3_CT/ICT
	14	3.52	352	0.1135	HOMO–3 → LUMO+2 (38), HOMO–1 → LUMO+2 (13)	ML_dapz-CF3_CT/ICT
	16	3.58	347	0.0388	HOMO–1 → LUMO+12 (21)	L_dapz-CF3_L_bpy_CT
	20	3.77	329	0.0310	HOMO–1 → LUMO+4 (22), HOMO → LUMO+4 (12)	ML_dapz-CF3_CT/L_dapz-CF3_L_bpy_CT

*^a^* Calculated on the level of theory of B3LYP/LANL2DZ/CPCM (solvent = CH_3_CN). *^b^* The actual percent contribution = (configuration coefficient)^2^ × 2 × 100%. *^c^* **L1** = L_dapz-OMe_, **L3** = L_dapz-CF3_.

## Data Availability

Not applicable.

## Supplementary Materials

The following supporting information can be downloaded at: https://www.mdpi.com/article/10.3390/ma16206792/s1, Figures S1–S8: ^1^H and ^13^C NMR spectra of **L1**, **L3**, **1**(PF_6_)_2_, and **3**(PF_6_)_2_; Figures S9–S11: MADLI-TOF mass spectra of **1**(PF_6_)_2_–**3**(PF_6_)_2_; Figure S12: HPLC spectra of **1**(PF_6_)_2_–**3**(PF_6_)_2_; Tables S1 and S2: Single-crystal X-ray data of complex **3**(PF_6_)_2_; Figure S13: Absorption and emission spectra of ligand **L1**–**L3**; Figure S14: Absorption, emission, and excitation spectra of **1**(PF_6_)_2_–**3**(PF_6_)_2_ in CH_3_CN; Figure S15: Emission spectra changes of **1**(PF_6_)_2_ and **3**(PF_6_)_2_ in different solvents; Figure S16: Emission spectra of **1**(PF_6_)_2_–**3**(PF_6_)_2_ in solid state; Figure S17: Emission spectra changes of **1**(PF_6_)_2_–**3**(PF_6_)_2_ at different excitation wavelengths; Figure S18: CVs and DPVs of ligands **L1**–**L3**; Figures S19 and S20: DFT calculation results of **1**(PF_6_)_2_ and **3**(PF_6_)_2_; Figure S21: TDDFT calculation results of **1**(PF_6_)_2_–**3**(PF_6_)_2_.
