# Supplementary material for: Dual-Emissive Monoruthenium Complexes of N(CH3)-Bridged Ligand: Synthesis, Characterization, and Substituent Effect
Source: Materials (Basel). 2023 Oct 20;16(20):6792. doi: 10.3390/ma16206792 (PMC10607950; doi:10.3390/ma16206792)
Supplement: Supplementary file 1 [file materials-16-06792-s001.zip › materials-2641895-supplementary.pdf]

Supporting Information for

Dual-Emissive Monoruthenium Complexes of $N(CH_3)$-Bridged Ligand: Synthesis, Characterization, and Substituent Effect

Si-Hai Wu,,† Zhe Zhang,† Ren-Hui Zheng,† Rong Yang,† Lianhui Wang,*,†*

Jiang-Yang Shao,‡ Zhong-Liang Gong,,‡ and Yu-Wu Zhong‡*

† School of Medicine, Huaqiao University, Quanzhou, Fujian 362021, China

‡ CAS Key Laboratory of Photochemistry, CAS Research/Education Center for Excellence in Molecular Sciences, Institute of Chemistry, Chinese Academy of Sciences, Beijing 100190, China

*To whom correspondence should be addressed. Email: wusihai@hqu.edu.cn; lianhui.wang@hqu.edu.cn; gongzhongliang@iccas.ac.cn

Table of Contents

NMR spectra of **L1**, **L3**, **1**(PF_6)$_2$, and **3**(PF_6)$_2$	S2–S5
Mass spectra of **1**(PF_6)$_2$, **2**(PF_6)$_2$, and **3**(PF_6)$_2$	S6
HPLC spectra of complexes **1**(PF_6)$_2$–**3**(PF_6)$_2$	S7
Single-Crystal X-Ray data of complex **3**(PF_6)$_2$	S7
Photophysical studies of these compounds	S8–S10
Electrochemical data of ligands **L1**–**L3**	S10
DFT/TD-DFT calculation results of these complexes	S11–S12

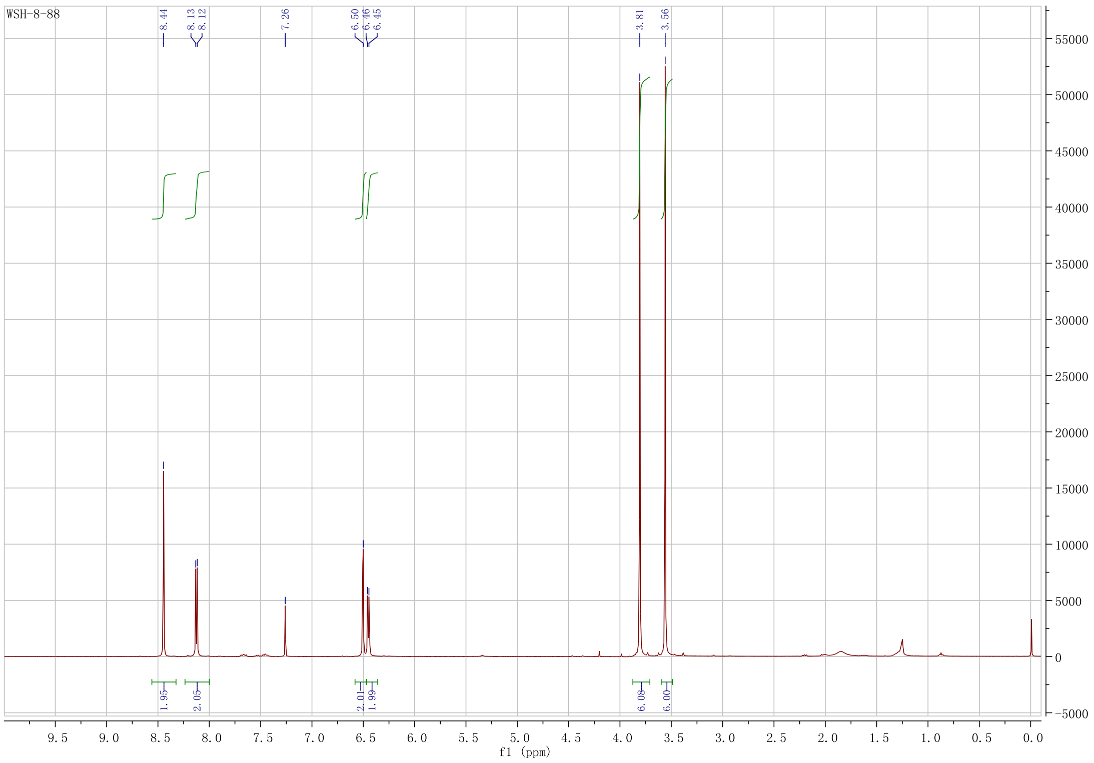

Figure S1. ^{1}H NMR spectra of **L1** in $CDCl_3$.

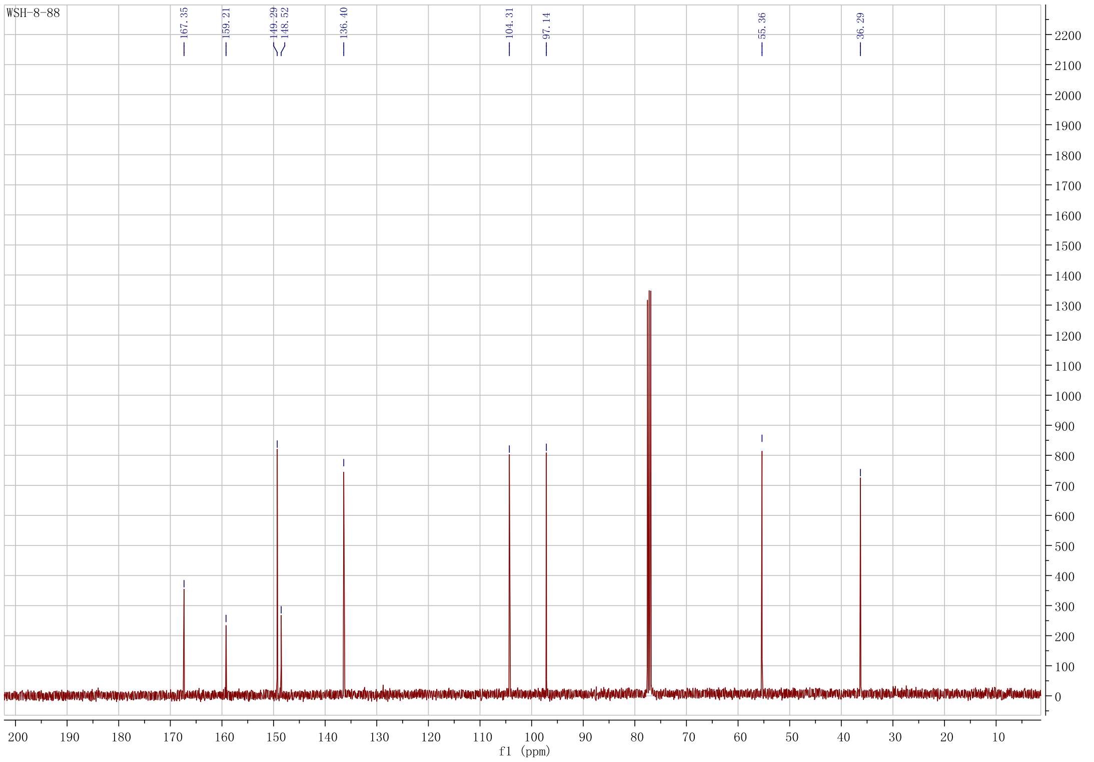

Figure S2. ^{13}C NMR spectra of **L1** in $CDCl_3$.

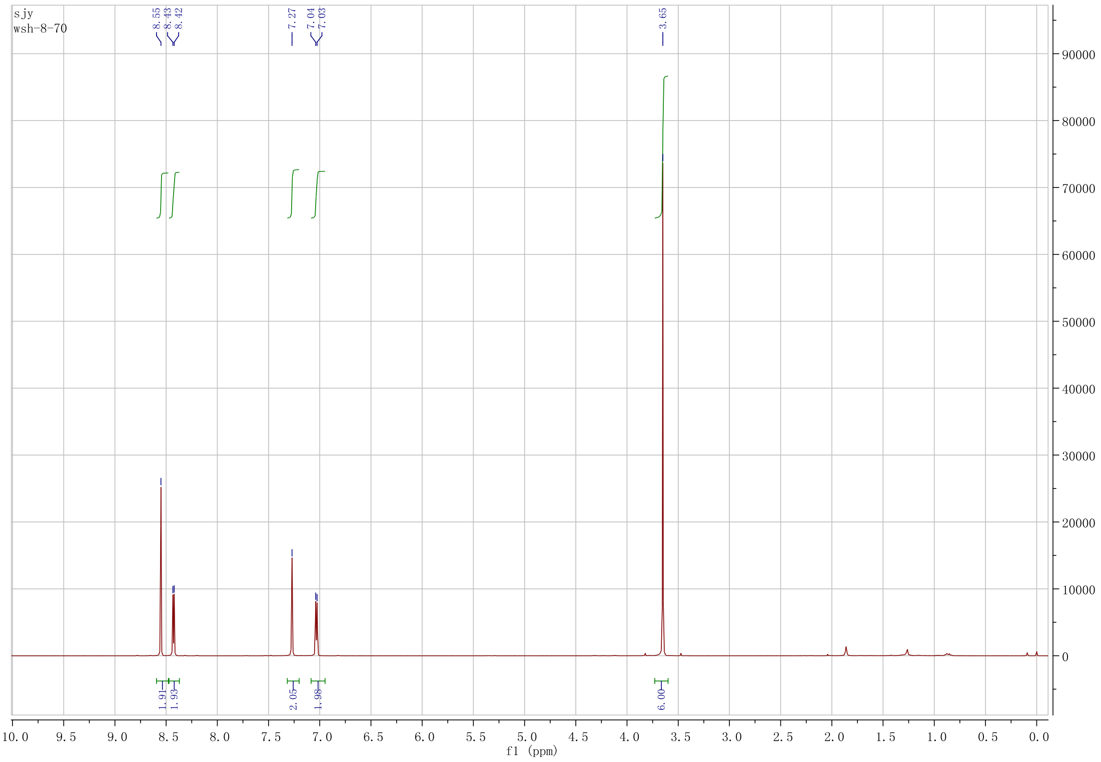

Figure S3. ^{1}H NMR spectra of **L3** in $CDCl_3$.

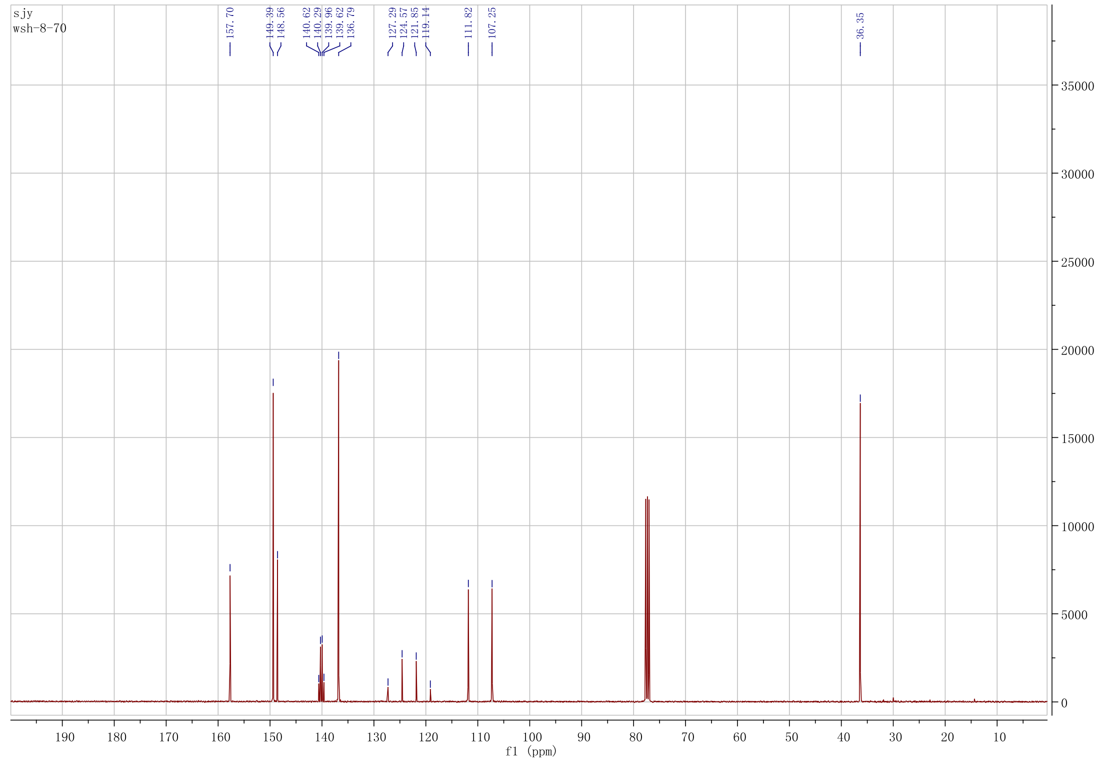

Figure S4. ^{13}C NMR spectra of **L3** in $CDCl_3$.

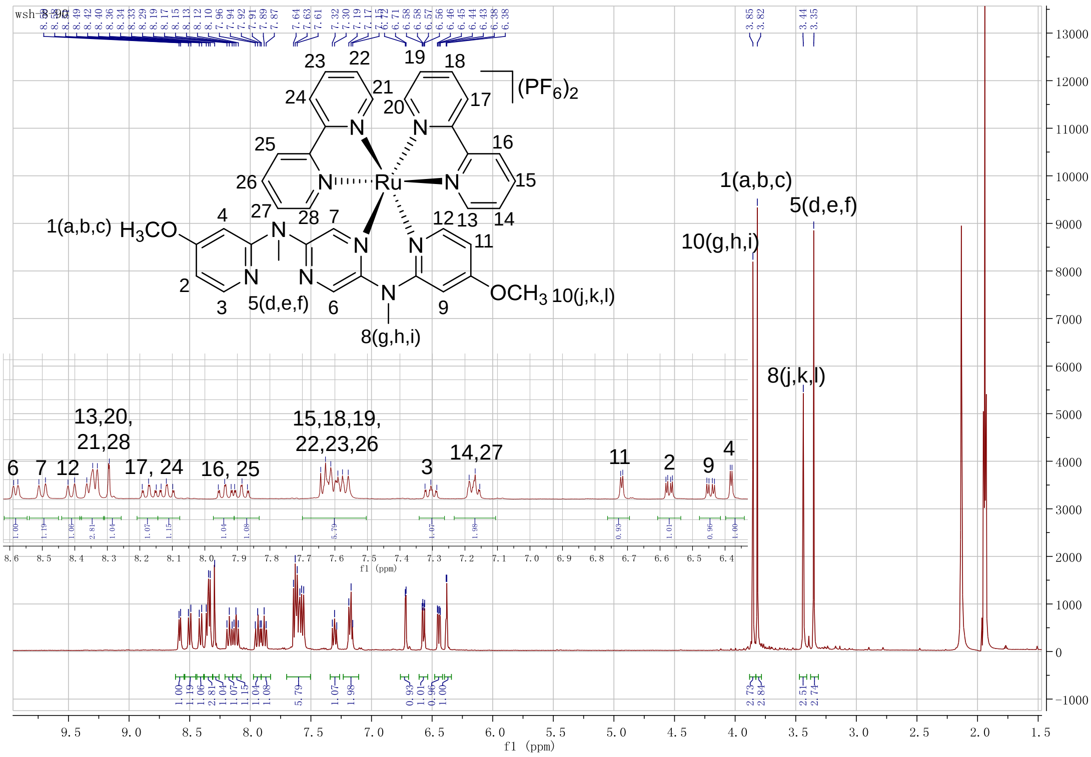

Figure S5. ^{1}H NMR spectra of **1**$(PF_6)_2$ in CD_3CN.

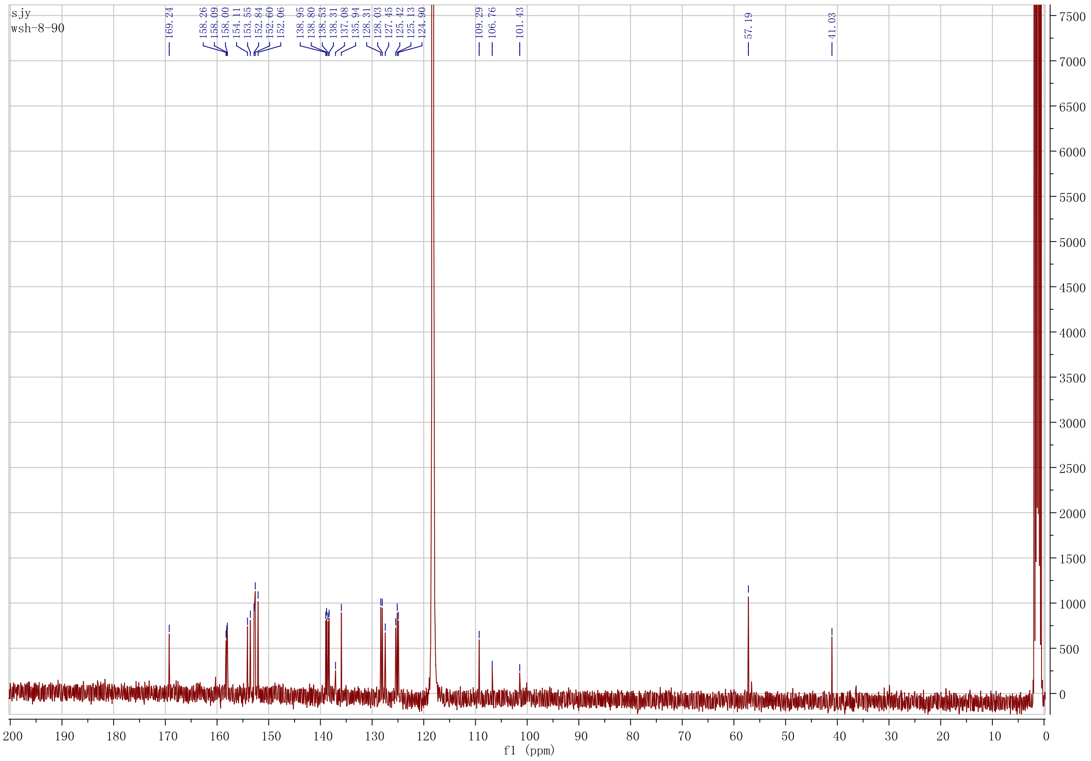

Figure S6. ^{13}C NMR spectra of **1**$(PF_6)_2$ in CD_3CN.

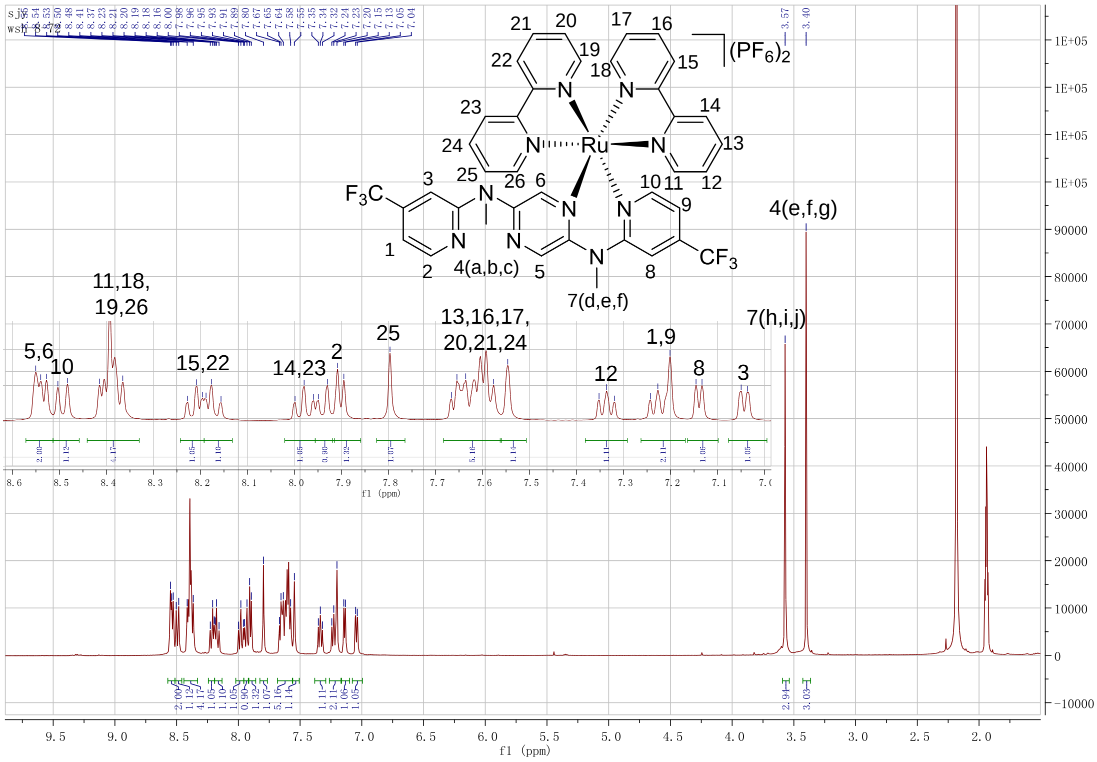

Figure S7. ^{1}H NMR spectra of **3**$(PF_6)_2$ in CD_3CN.

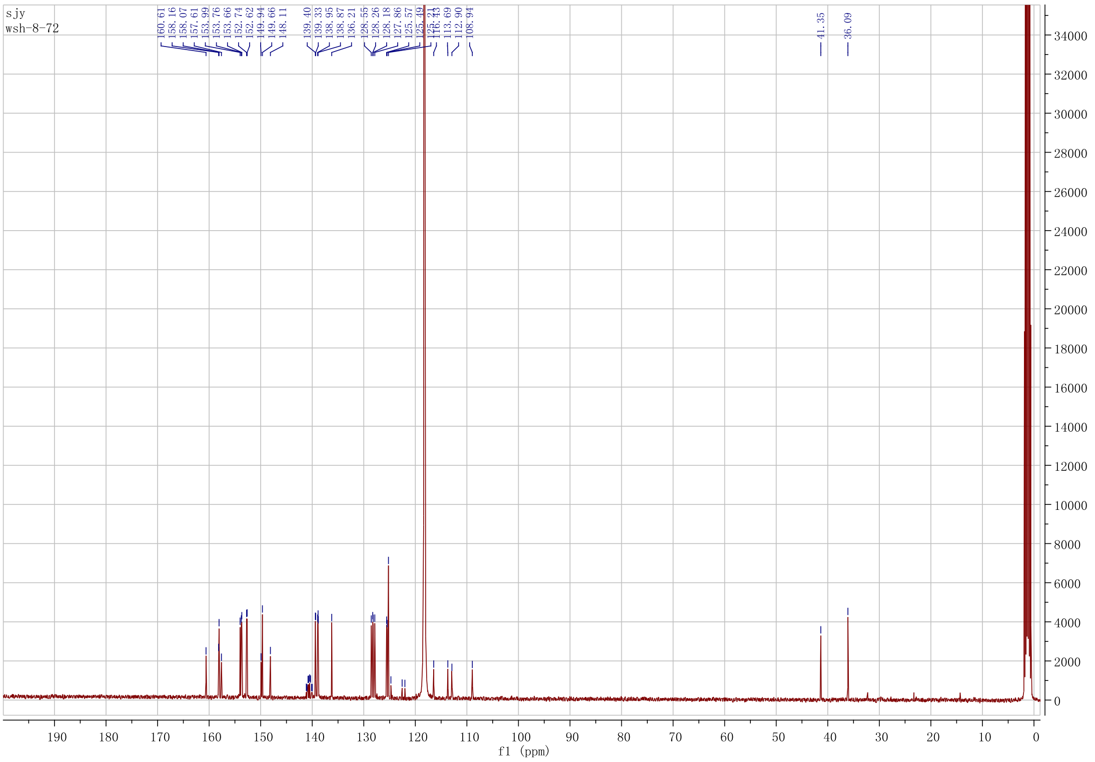

Figure S8. ^{13}C NMR spectra of **3**$(PF_6)_2$ in CD_3CN.

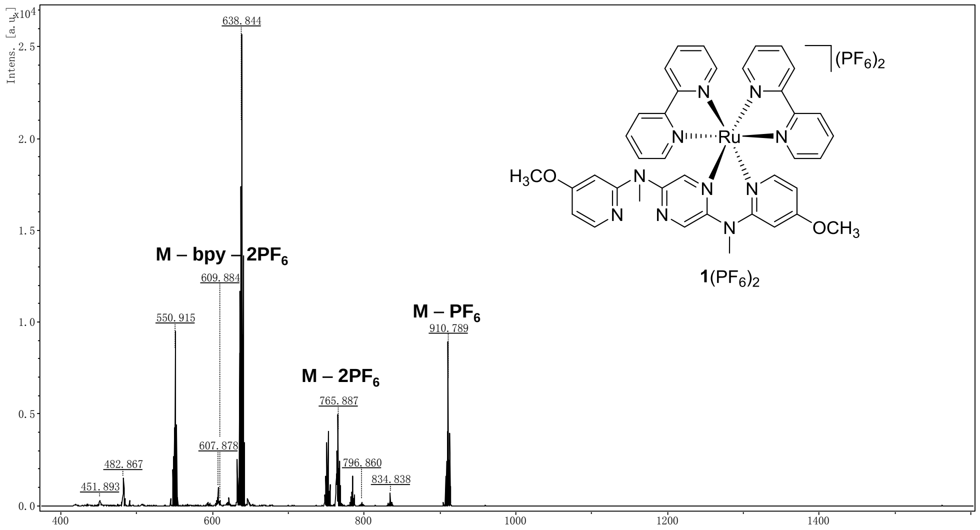

Figure S9. MADLI-TOF mass spectrum of **1**(PF_6)$_2$.

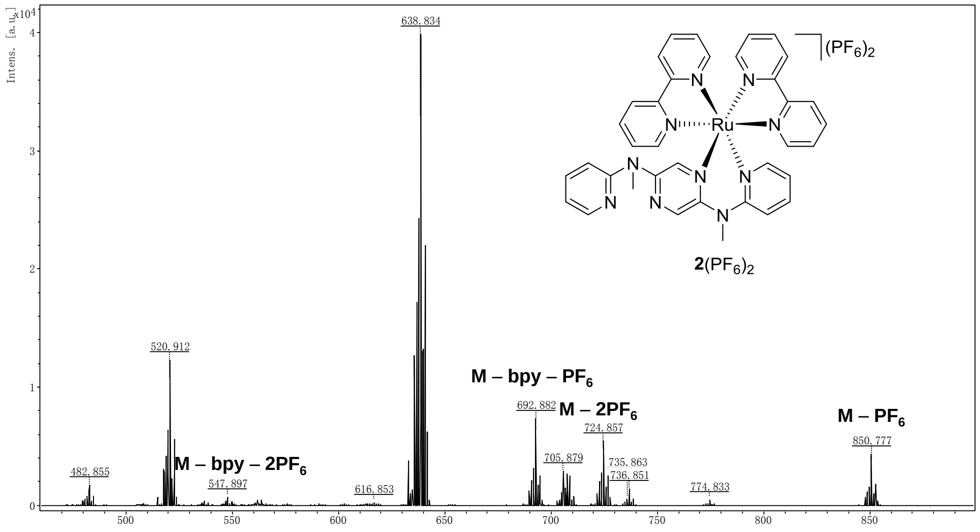

Figure S10. MADLI-TOF mass spectrum of **2**(PF_6)$_2$.

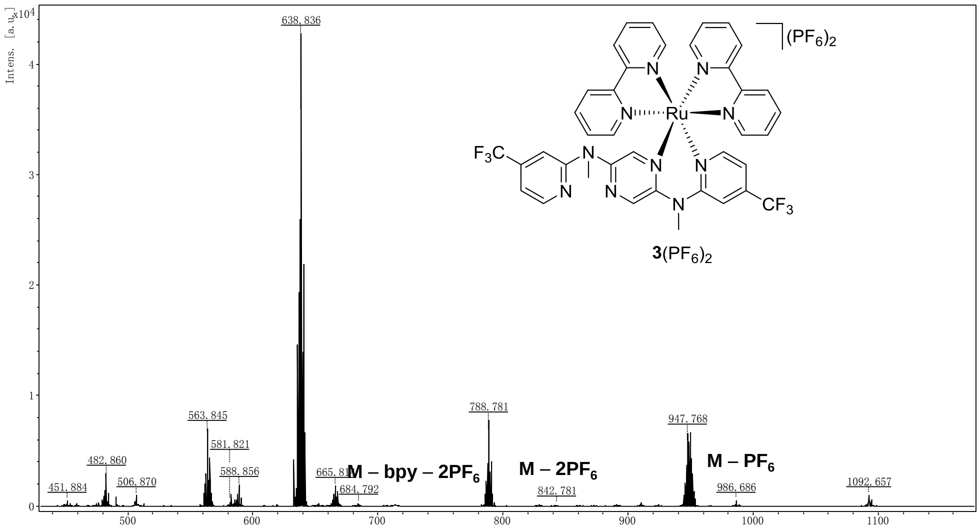

Figure S11. MADLI-TOF mass spectrum of **3**(PF_6)$_2$.

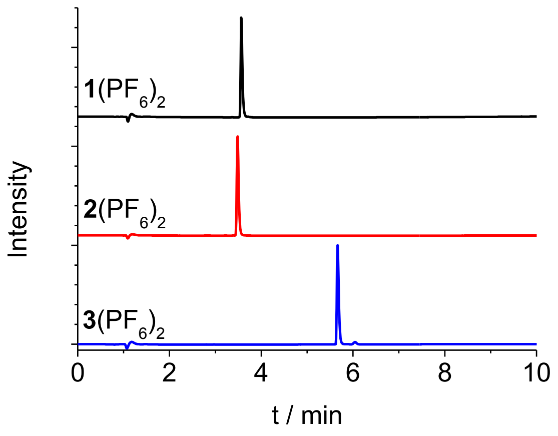

Figure S12. Analytical HPLC spectra of **1**$(PF_6)_2$ (black line), **2**$(PF_6)_2$ (red line), and **3**$(PF_6)_2$ (blue line). The noise peaks around 1.1 s are due to eluent solvents.

Table S1. Single-crystal X-ray data of complex **3**$(PF_6)_2$.

Compound	**3**$(PF_6)_2$
CCDC number	2107266
Empirical formula	$C_{38}H_{30}F_{18}N_{10}P_2Ru$
Formula mass	1131.73
Crystal system	Triclinic
a [Å]	11.6715(4)
b [Å]	13.8183(4)
c [Å]	14.1803(4)
V [Å^3]	2107.13(12)
α [°]	74.878(3)
β [°]	74.737(3)
γ [°]	78.194(2)
Z	2
Density [Mg·cm^{-3}]	1.784
*R*1 (final)	0.0477
*wR*2 (final)	0.1105
*R*1 (all)	0.0585
*wR*2 (all)	0.1155

Table S2. Selected bond lengths (Å) and angles.

Complex	Bond lengths (Å)		Bond angles	
**3**$(PF_6)_2$	Ru(1)–N(1)	2.056(3)	N(1)–Ru(1)–N(6)	78.86(11)
	Ru(1)–N(2)	2.078(3)	N(2)–Ru(1)–N(3)	78.68(11)
	Ru(1)–N(3)	2.062(3)	N(4)–Ru(1)–N(5)	88.30(10)
	Ru(1)–N(4)	2.068(3)	N(2)–Ru(1)–N(4)	91.11(10)
	Ru(1)–N(5)	2.099(3)	N(2)–Ru(1)–N(5)	97.14(10)
	Ru(1)–N(6)	2.071(3)	N(1)–Ru(1)–N(2)	96.38(11)

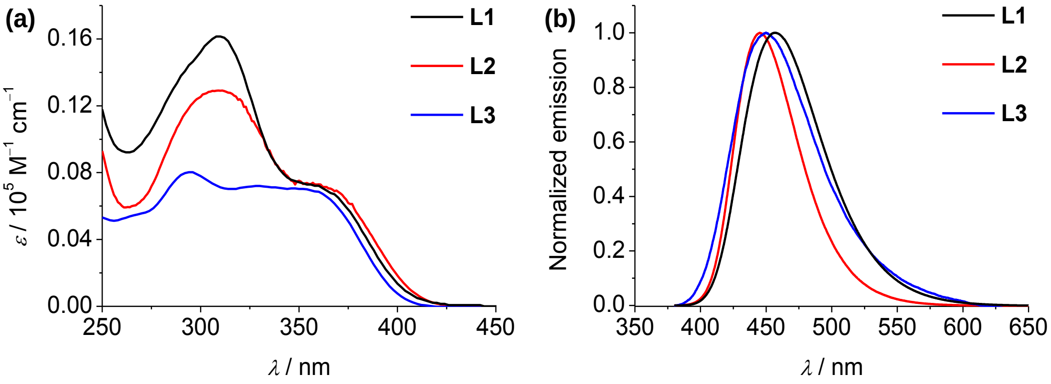

Figure S13. (a) UV/vis absorption spectra of ligand **L1**–**L3** in acetonitrile. (b) Emission spectra of ligand **L1**–**L3** in acetonitrile on excitation at 360 nm.

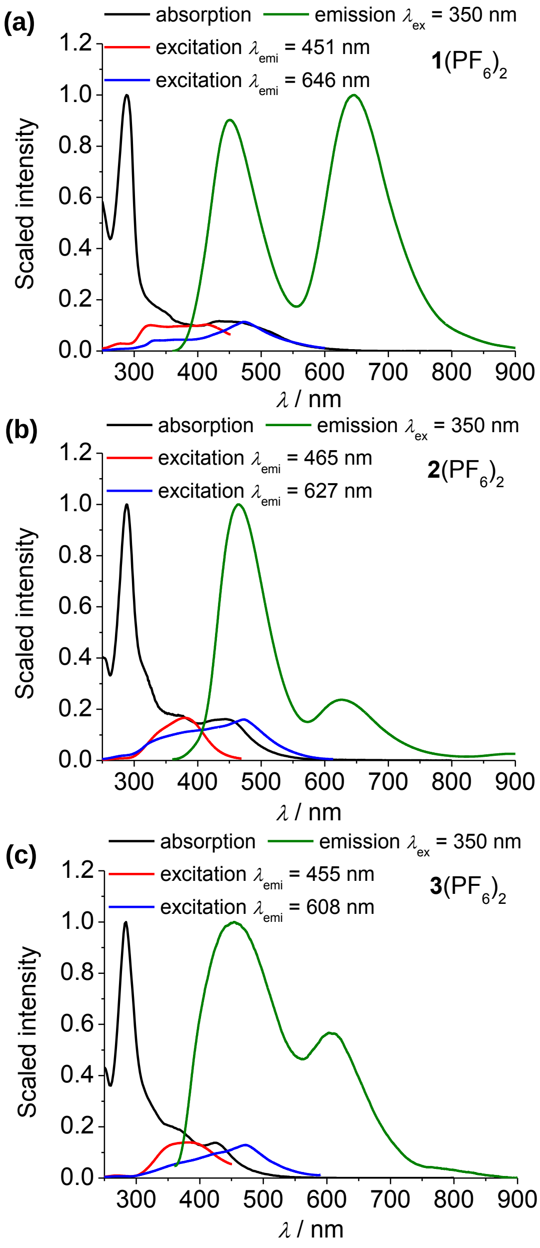

Figure S14. Absorption, excitation, and emission spectra of **1**(PF_6)$_2$ (a), **2**(PF_6)$_2$ (b), and **3**(PF_6)$_2$ (c) in CH_3CN.

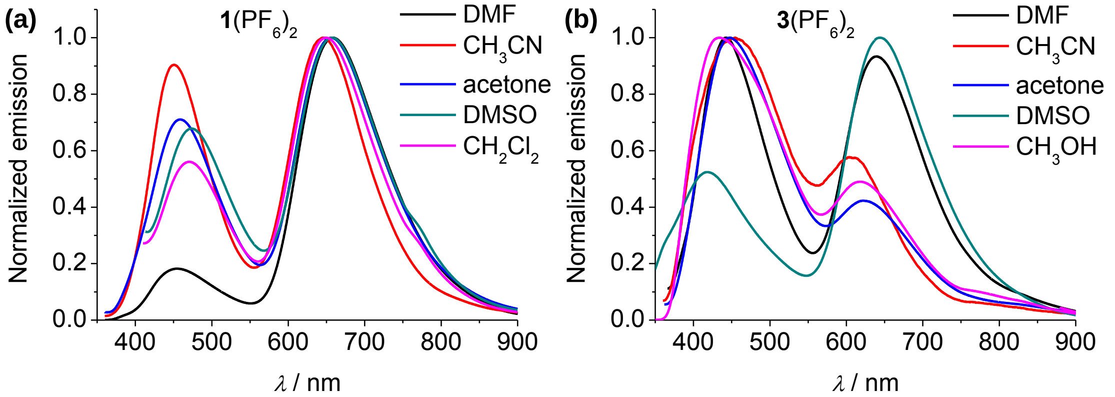

Figure S15. Normalized emission spectra changes of **1**(PF_6)$_2$ (a) and **3**(PF_6)$_2$ (b) in different solvents.

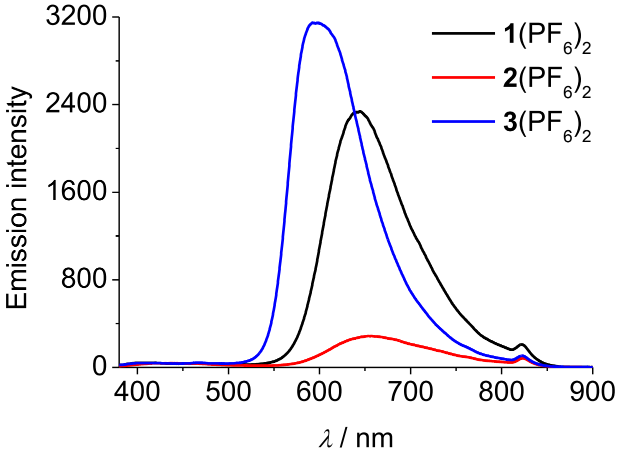

Figure S16. Emission spectra of **1**(PF_6)$_2$ (a), **2**(PF_6)$_2$ (b), and **3**(PF_6)$_2$ (c) in solid state on excitation at 350 nm.

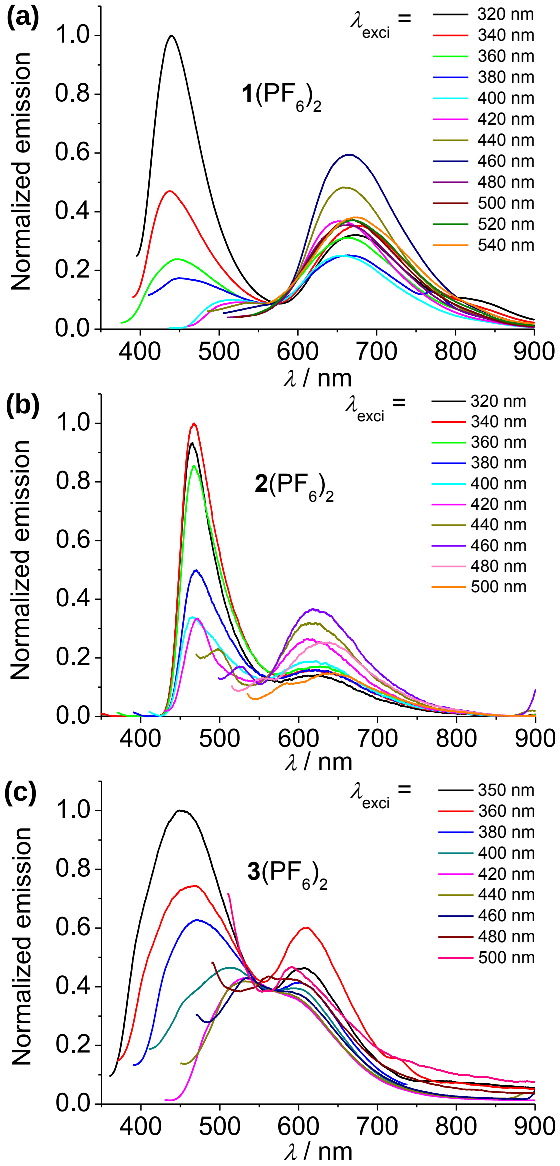

Figure S17. Emission spectra changes of **1**(PF_6)$_2$ (a), **2**(PF_6)$_2$ (b), and **3**(PF_6)$_2$ (c) in CH_3CN at different excitation wavelength.

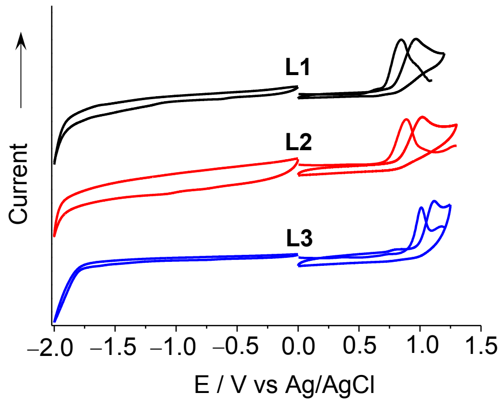

Figure S18. CVs and DPVs of ligands **L1**–**L3** in acetonitrile containing 0.1 M Bu_4NClO_4 at a scan rate of 100 mV/s. The working electrode is a glassy carbon, the counter electrode is a Pt wire, and the reference electrode is Ag/AgCl in saturated aq. NaCl.

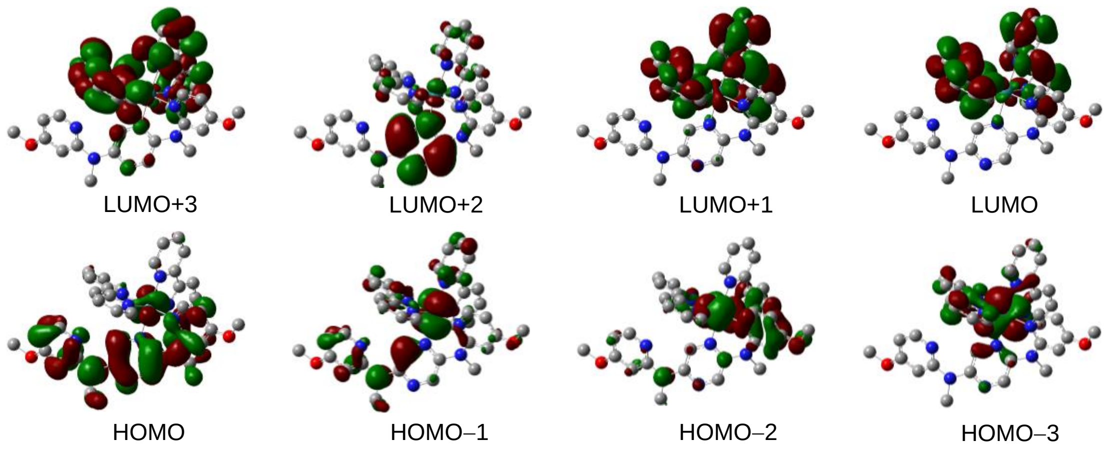

Figure S19. Isodensity plots of selected frontier molecular orbitals of complex **1**$^{2+}$. All orbitals were computed at an isovalue of 0.02 e bohr^{-3}.

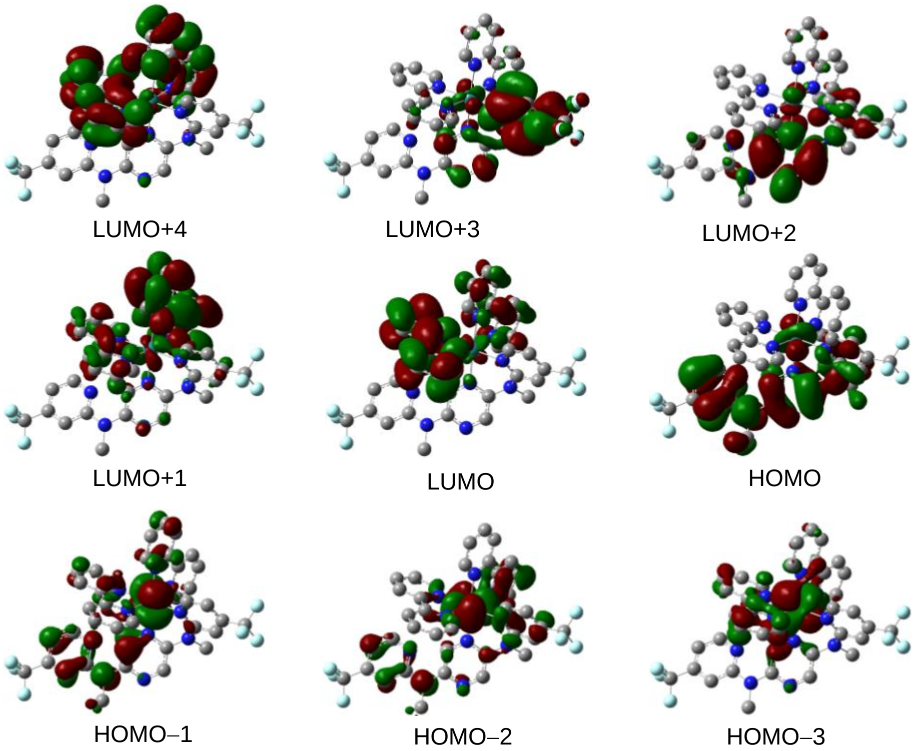

Figure S20. Isodensity plots of selected frontier molecular orbitals of complex **3**$^{2+}$. All orbitals were computed at an isovalue of 0.02 e bohr^{-3}.

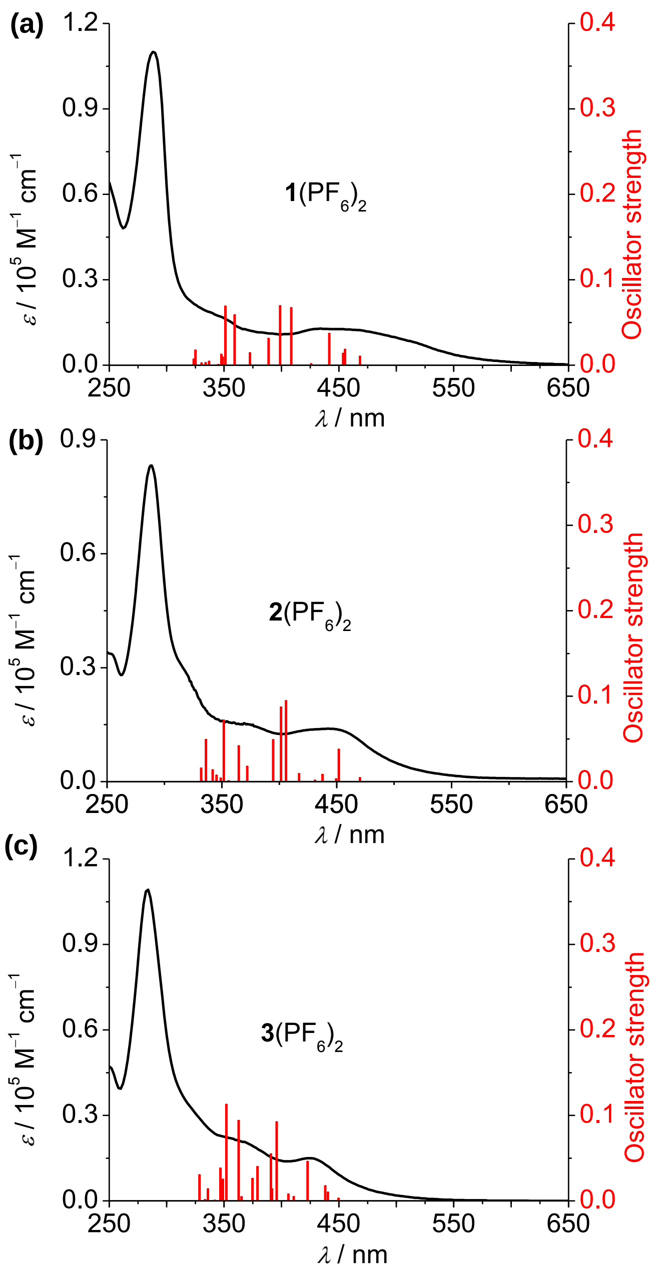

Figure S21. TDDFT-predicted excitations (red lines) of (a) $\mathbf{1}^{2+}$, (b) $\mathbf{2}^{2+}$, and (c) $\mathbf{3}^{2+}$. The absorption spectra of **1**$(PF_6)_2$–**3**$(PF_6)_2$ (black curves) are included for comparison.
